# The Blessed Clay from Boho, Northern Ireland: Can the Nature of Spiritual Healing Sites Guide us Towards New Sources of Drug Discovery?

**DOI:** 10.1007/s10943-025-02311-9

**Published:** 2025-04-19

**Authors:** Gerry A. Quinn, Drake Harris

**Affiliations:** 1 Institute of Biomedical Sciences, Ulster University, Coleraine, Northern Ireland, UK; 2 Center for Environmental Research and Earth Sciences (CERES), Salem, MA 01970 USA

**Keywords:** Spiritual healing sites, Medicinal bioprospecting, *Streptomyces*, Soil

## Abstract

In 2016, a group of scientists isolated *Streptomyces* sp. myrophorea, an antibiotic producing microorganism, from the “Blessed Clay” of Fr. McGirr, an old Irish folk cure found in Boho, County Fermanagh, Northern Ireland. In this essay, the authors examine the common elements between this spiritual healing site and others around the world and compare these motifs with characteristics commonly associated with the discovery of antibiotic producing microorganisms. The authors also contemplate whether this discovery adds any credence to spiritual healing practices and if sites like these have the potential to guide researchers toward new pharmaceutical discoveries. Taking into account local sensitivities and cultural traditions, the authors propose that similar spiritual healing sites should be carefully studied for evidence of microorganisms that might produce useful pharmaceuticals.

## Introduction

Boho (pronounced Bo) is a small farming area in the south of County Fermanagh in Northern Ireland situated close to the border with the Irish republic. It is thought that this area has been inhabited since Neolithic times given the ample evidence of ancient monuments in the area (Wakeman, 1875). Besides its remote location, the area has generally been forgotten in terms of investment and development since the downgrading of the main arterial road from Belfast and the closure of the railway to the nearby town of Enniskillen in the 1950s. However, this neglect may have been a blessing in disguise, since the area retains one of the highest floristic diversities and field densities in Northern Ireland (Cooper et al., 2003). Perhaps of equal significance or as a consequence of its poor developmental status, this same isolation may have likewise preserved its unique ethnopharmacological traditions (Ballard, 2009). These reputedly originated from the druids (Wagner et al., 2020) and are generally referred to as “the cures” (Foley, 2015). These cures are passed down from parents to the most reliable offspring who are able to respect their traditions. In some exceptions, these cures may be commonly known; however, the specifics of how these cures are constituted or applied may be vague, perhaps as a consequence of having no recognized guardian of their tradition (Foley, 2015).

An important example of one such “cure” is the soil that covers the grave of a cleric who died over 200 years ago, Father James McGirr (1745–1815). Fr. McGirr was one of the first priests to emerge from years of religious oppression in Ireland as a result of the Penal laws. These laws effectively meant that regular Catholic clergy and Bishops were banned from Ireland and could be hanged if they returned. The remaining secular priests were required to make an oath to the British crown but many refused and went into hiding (Bishop, 2016; O’Connor, 2018). In addition, Catholics were banned from voting, holding public office, owning land, teaching and attending their own services (O’Connor, 2018). During this time priests would resort to conducting religious ceremonies in secret in countryside areas, perhaps at the entrances to caves or other so called “mass rocks”, which are still a familiar feature of the Boho landscape (Bishop, 2016; Donnelly, 2004).

Fr McGirr was known as an active practitioner of the traditions of the local healers in countryside area of Boho and when he was advancing in years his parishioners were worried that there would be nobody to take his place. However, he reassured his flock saying that the soil that covered him would also do the same healing as he did when he was alive. Soon after, although the exact date is not recorded, a tradition developed of people taking a small part of the soil from the top of this cleric’s grave. It is not generally thought that this soil became more miraculous when he died because he had stated that the soil that covered him would do ‘*the same’* healing as it had done when he was alive but as a reference to the healing properties of the local soil. The soil would be wrapped in a piece of cloth by the pilgrims and taken home. The person would not talk to anyone on the way home and once there they would put the package of soil underneath their pillow for 3 or 4 days. The next day they would return the soil to the grave (Terra et al., 2018). Some would also use the soil for toothache and place the cloth package in their mouths. Presently, this cure is neither officially nor unofficially endorsed by the local church; however, church authorities have erected signs with instructions on appropriate behavior for pilgrims to this graveyard since it is still in current use.

This particular ethnopharmacological tradition might have disappeared with the last generations who practiced it, if it was not for an international group of scientists who (at that time) were searching for new sources of antibiotics and other medicines due to shortages in the approval of new antibiotics and rising reports of antibiotic resistance (Aminov, 2010; Terra et al., 2018).

One relatively new strategy in the search for new antibiotics adopted by these scientists is the exploration of extreme or unusual environments, such as deserts, hot springs or areas of extreme acidity/alkalinity (Abdelkader et al., 2018; Chen et al., 2022; Tiago et al., 2004). The rationale behind this strategy is that physiological adaptations of antibiotic producing microorganism to extreme or unusual environments may result in equally novel compounds or even ways of producing these. It is hoped that these new structures might have the necessary degree of structural dissimilarity from older generations of antibiotics to mitigate the development of antibiotic resistance (Sivalingam et al., 2019). Some of these physiological adaptations have already been observed in microorganisms isolated from the Lindze desert in China, which use more efficient translation systems to produce antibiotics (Chen et al., 2022).

Floristically, much of the uplands of Boho and the West Fermanagh Scarplands are classified as calcareous grassland, which constitutes a rare habitat type in Northern Irish terms since it represents less than 1% of the total land area (Cooper et al., 2003). It is in this habitat that new *Streptomyces* spp. were identified that produce antimicrobial, antiviral and antifungal compounds (Quinn et al., 2021a, 2021b; Terra et al., 2018). Perhaps coincidentally or not, these soil samples are also associated with local spiritual and ethnopharmacological healing traditions.

Therefore, the discovery of the Blessed Clay of Fr McGirr provides an opportunity to compare the common themes of local spiritual traditions as they pertain to “the cure” and common motifs in medicinal bioprospecting for organisms that produce much needed replacement pharmaceutical products. This comparison may help scientists identify potential prospects for future pharmaceutical investigations.

## Methods

The following narrative review is based on analysis of literature identified through searches of PubMed and Google Scholar in relation to the twin themes of healing practices linked to soils and the identification of microorganisms that produce chemotherapeutic compounds.

## Common Spiritual Based Motifs Around the Blessed Clay in Boho

### Sacred Soils

Perhaps one of the most familiar associations between soil and healing in Ireland might be from the Christian tradition which recounts that Jesus spat on the soil, made a paste and rubbed it on the eyes of a blind man, restoring his sight (John 9:6–7). Indeed, the Blessed Clay of Fr McGirr is not unique in terms of spiritual traditions but rather it shares many similarities with other practices in Ireland and even around the world.

**Fig. 1:**
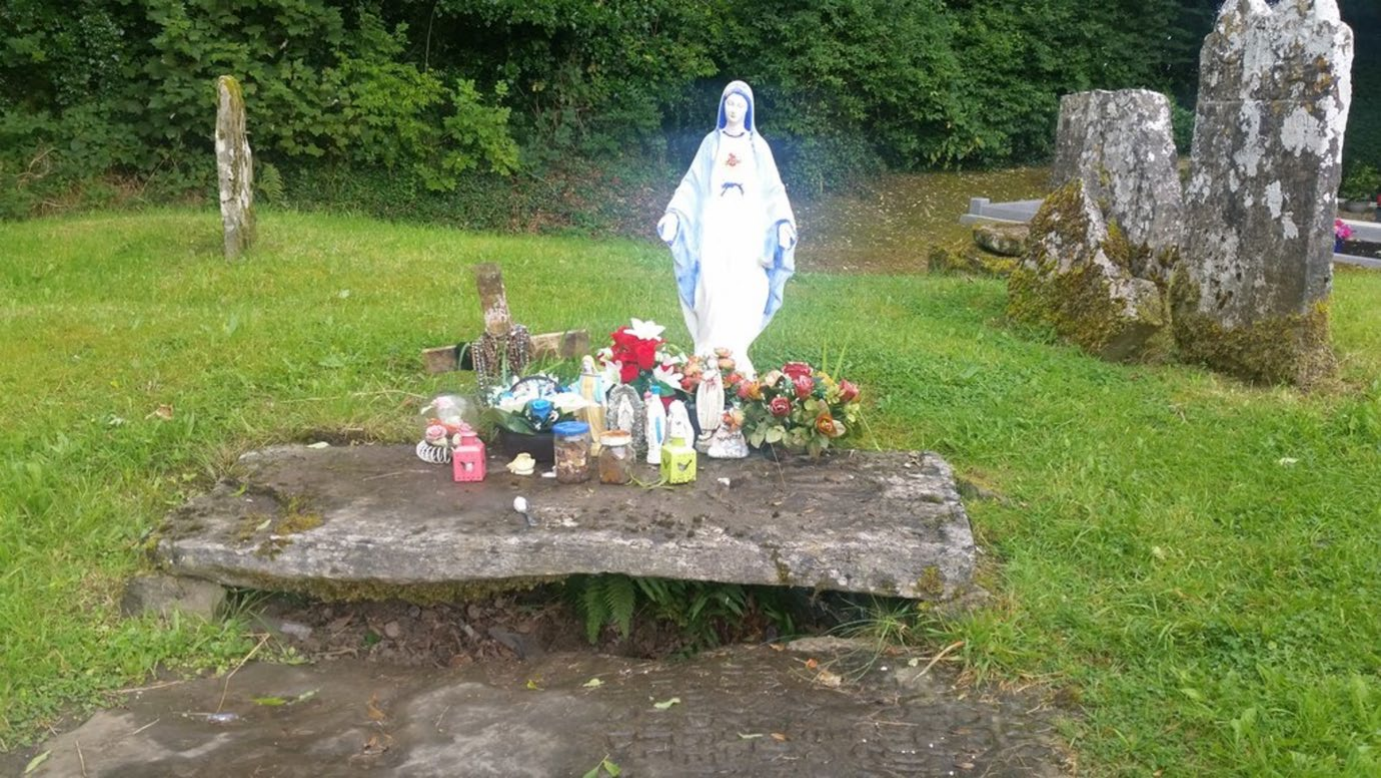
The Grave of Fr. Peter Smith in Old Kill Graveyard Co. Cavan, Republic of Ireland. Photo © Louise Price (cc-by-sa/2.0) (19 August 2016)

In Co. Cavan (Irish republic) which is just across the border from Co. Fermanagh, soil from the grave of Fr. Peter Smith (a healing priest who lived in the early 1800s), in Kill graveyard, is associated with healing of various illnesses. People are instructed to take a very small portion of clay, wrap it in cloth and sleep on it for 3 nights. On 4th day, the soil is returned to grave. In a variation of these instructions, the soil is returned to the grave but the cloth in which it is wrapped is tied to a bush which grows beside the grave (Gaffney, 1930) (Fig. 1).

Soil from a grave on St. Mogue’s Island, in the nearby Templeport Lake, also in Co. Cavan, is also reputed to be a source of protection. In this instance a small portion of soil is collected and sometimes sewn into people’s garments as an insurance against fire or drowning (Glassie, 1982).

Similar practices are found in other continents such as the soil from the Santuario de Chimayó, in New Mexico, USA. Here, pilgrims gather soil from a hole in a church floor which is reputed to heal a variety of illnesses. This practice is thought to have been taking place since the beginning of the nineteenth century and possibly much longer (Hendrickson, 2018). The Santuario de Chimayó is thought to be built on top of a hot spring which is no longer active but was used by previous generations of Tewa Indians as a healing place (Johnson, 2023).

In Houthem, the Netherlands, there have also been reports of healing limestone powder from the grave of St Gerlachus. This powder was previously used for healing humans but is now restricted to use on animals, plants and flowers (Weertz et al., 2020). Similarly, in Haillot, Belgium, soil from the grave of St Mort is also used for healing, especially for conditions such as gout and toothache (Weertz et al., 2020).

An even older traditional exists in British Columbia, Canada, where the Helsuit people routinely use an ancestral glacial clay from Kisameet Bay against many diseases. The medicinal potential of this clay was verified by Prof. Julian Davies and his team at the University of British Columbia, Canada. Their research demonstrated that a small amount of the clay was enough to kill an array of pathogenic bacteria commonly found in clinical settings (Behroozian et al., 2016).

The authors of this discussion acknowledge that clays are often used around the world with no particular spiritual traditions or intentions (Spielvogel et al., 2021). However, in these cases, the healing properties are usually attributed to the physiochemical nature of the soils and not to any particular microorganisms living in the soil that might produce pharmaceuticals.

It is also not our intention to detract from the utility of faith based healing practices or the firmly held beliefs of those that practice them but perhaps the idea that many cultures and spiritual traditions around the world focus on particular types of soils may suggest a unifying scientific answer. After all, a large amount of antibiotics in clinical use today were originally derived from soil based microorganisms (Donald et al., 2022).

There are other common motifs which are related to the transport of healing soils, such as the use of cloth which has been reported in several cures, however, we have been unable to ascertain any connections that might relate this to any medical applications or antimicrobial producing microorganisms (Gaffney, 1930; Quinn, 2023; Terra et al., 2018).

### Similar Motifs Between Spiritual Healing Sites and Areas that are Commonly Associated with Discovery of Antibiotic Producing Microorganisms

We will now discuss common motifs associated with spiritual healing sites such as the Blessed Clay in Boho and the discovery of antibiotic or medicinal producing organisms mainly from the genus *Streptomyces*. Although this may seem like a tangential leap in subject matter, the majority of modern clinical antibiotics are derived from soil dwelling *Actinobacteria* or *Streptomyces* (Donald et al., 2022). Indeed, one of the first antibiotics that was effective against tuberculosis (streptomycin) was isolated from a soil sample from Rutgers farm in upstate New York (Schatz et al., 1944). Since then scientists have used similar methods of discovery to isolate many of the well-known clinical antibiotics (Donald et al., 2022).

### Poor Area, Strong Beliefs

One common motif that links the Blessed Clay with other sites of spiritual healing around the world is the low soil fertility. Historically, the upland area in Boho was classified as agriculturally poor by the British crown surveyors sent to Ireland in the seventeenth century. This is because the Boho grasslands are composed of thin soils on top of a limestone bedrock interspersed with bog, heath and fen meadow (Cooper et al., 2003). Economically, this is also a relatively poor farming area, with many part-time farmers who rely on other occupations as the main source of their income while maintaining their farming heritage (Cruickshank, 2017).

In addition to this, the area of Boho is traditionally an area of high religious adherence. However, this might not just indicate that there are numerically more people with some sort of spiritual life, it could also mean that these people are more conditioned to be more aware of those things or events that might be connected to spiritual manifestations. That is to say, they might be more likely to pay attention to reports of spiritual cures.

Of course, this could be said of many other spiritual healing sites, were people of poor lands and strong faith perceive or accept a healing practice or phenomenon that seems to have a spiritual dimension such as sites at Lourdes (France), Fatima (Portugal) and the Santuario de Chimayó (USA) (Schienle et al., 2020).

### Poor Soils and Isolation of Antibiotic Producing Organisms

Interestingly, antibiotic producing species of *Streptomyces* are also associated with nutrient poor soils, caves and general limestone features (karst) (Yucel & Yamac, 2010). Although these *Streptomyces* can be easily outcompeted by faster growing “mesophilic” microorganisms for nutrients in fertile areas, these antibiotic producing bacteria have a competitive advantage in areas of high physiological stress such as arid areas or hot springs (Shi et al., 2017).

Other sites around the world that contain poor soils on top of limestone environments have similarly been associated with spiritual healing such as those at Lourdes (France), Medjugorje (Croatia) and Bom Jesus da Lapa Religious Shrine (Brazil) (François et al., 2014; Rossi et al., 2020). Indeed the karst environment has proven to be a rich source of Streptomycetes (Maciejewska et al., 2016; Yucel & Yamac, 2010) including discoveries in some in some of the oldest known human settlements (Hamedi et al., 2019) and even in areas as far flung as Bhutan where limestone aquifers and caves are considered to be of spiritual significance (Allison, 2019).

### Life Cycles

Another motif symbolically associated with the Blessed Clay is that of the cycle of life. Traditionally, periods of Irish history are referred to as cycles such as the Ulster cycle, the Fenian cycle and the King cycle. Indeed, these traditions relate to the cyclical nature of life and death and rebirth, characteristically incorporated into Celtic artistic styles. The Blessed Clay itself comes from outside a church which is associated with the lives of the people, and is surrounded by a graveyard which represents the final destination or death. Indeed, graves in many traditions also symbolize the gateways to the next life or the underworld. In Irish mythology, caves and burial mounds were the entrance to the other world of the immortals, the Tuatha Dé Danann, who were immune to aging and sickness (Macritchie, 1902). This is very interesting specifically for an area such as Boho which has an abundance of limestone caves. Even the townland of the Sacred Heart Church in Boho where the Blessed Clay is located (Reyfad) contains some of the largest and deepest caves in Ireland such as the Reyfad—Pollnacrom—Polltullybrack cave system (Mitchell, 1987).

**Fig. 2:**
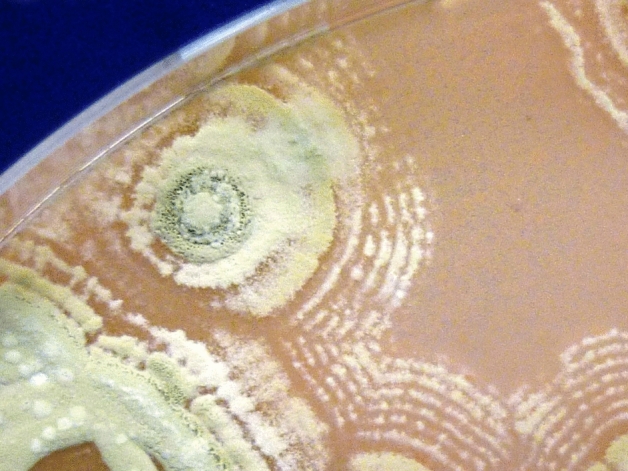
*Streptomyces* sp. myrophorea at sporulation stage. Image by Youngbohemian, license: CC BY-SA 4.0 (created 19 March 2016)

Of course, karst areas also have a long association with the successful isolation of *Streptomyces* perhaps due to their high pH and nutritionally poor soils (Axenov-Gibanov et al., 2016; Yucel & Yamac, 2010). The motif of the cycle of life, death and rebirth is also reflected in the life cycle of *Streptomyces* itself*.* During the ‘final flourishes’ of its growth, *Streptomyces* produce secondary metabolites which include antibiotics and spores which will form the next generation. In doing so, these *Streptomyces* sacrifice their own cells, releasing nutrients for the next generation in a process called autophagy. Macroscopically, these growth cycles can look like a series of concentric circles rather like those produced by mushrooms on a grass lawn (Fig. 2).

One of the key aspects to the production of antibiotics by *Streptomyces* is the stimulus or trigger. Molecularly, this production is coordinated by biosynthetic antibiotic gene synthesis clusters, some of which are known as silent or cryptic which means that these genes are not always active but can be stimulated by certain cues (Hoskisson & Seipke, 2020). These triggers can be induced by many things including physiological stress, limited nutrients, competitors or even the presence of a specific mineral (Hoskisson & Seipke, 2020). This means that it is not guaranteed that environmental *Streptomyces* isolates will produce antibiotics under all conditions. These events may have similarities with spiritual healing sites in that it is never guaranteed that any healing will occur.

### Continuity of Healing Across Several Spiritual Traditions

**Fig. 3:**
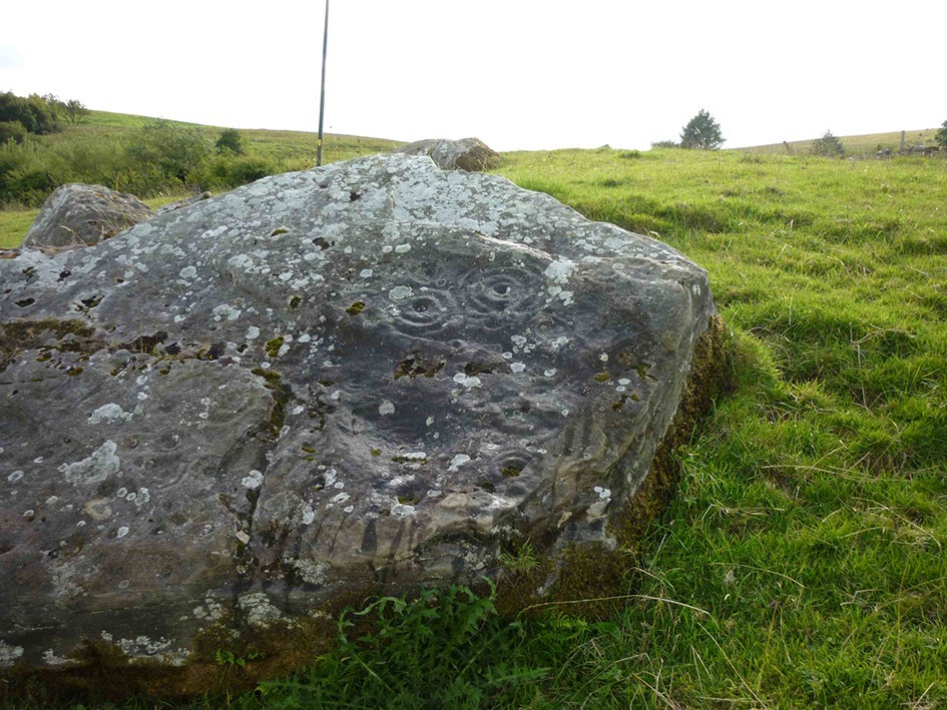
One of the six Reyfad stones with characteristic cup and ring markings. Image by Youngbohemian, license: CC BY-SA 3 (Created: 15 August 2010)

The Blessed Clay of Fr McGirr is located outside the present day Sacred Heart Church, which according to legend, has its origins with St Faber (Donnelly et al., 2003). The church fell into ruin during the disillusion of the monasteries and was reconstructed after the penal laws around (1832) (Donnelly et al., 2003). However, the use of the physical site where the Blessed Clay is found can be traced back further as the present church is built of the ruins of a pre-Christian site (Halpin & Newman, 2009). There is further evidence that the general area was also used as a ceremonial space by a series of six carved stones with cup and ring markings referred to as the Reyfad stones (Fig. 3) (Nash & Mazel, 2022). Although the exact age of these stones is not known, they are thought to be of a similar age to those found at Newgrange in the Irish republic (Eriksen, 2008; Wakeman, 1875). It is not unusual to find that modern religious sites are located in close proximity to older spiritual traditions. In Ireland it has even been reported that many of the ‘mass rocks’ used during Penal law times were situated on or near monuments from older pre-Christian traditions (Bishop, 2016). In USA, the Santuario de Chimayó is thought to be built on the remnants of a hot spring previously used by the Tewa Indians as a source of healing (Hendrickson, 2018; Johnson, 2023). It would be interesting to establish whether other spiritual healing sites had a more extensive history of use.

### Isolating Streptomycetes from Spiritual Sites

*Streptomyces* are frequently isolated from graveyards and other spiritual sites. The main subject of this paper details the discovery of a new species of *Streptomyces* which was associated with an ancient Irish spiritual tradition, that is, the Blessed Clay at Boho (Terra et al., 2018) identified from graveyard soil. However, there have also been other reports from around the world of *Streptomyces* isolated from other spiritual sites. For example, *Streptomyces cinerochromogenes* isolated from soil from the Tama Graveyard in Tokyo, Japan produces the antibiotic cineromycin B. ***Streptomyces lavendulae*** subsp. ***grasserius*** (Ueda et al., 1955) isolated from Tama graveyard produces the antibiotic grasseriomycin. Further discoveries include *Streptomyces parvus* strain C05 isolated from the walls of a Roman tomb located in Carmona, Seville, Spain, which produces the antibiotic and the anticancer compound granaticin (Gonzalez-Pimentel et al., 2019).

## Health and Spirituality

Although the Blessed Clay is traditionally believed to be the parting gift from the departed Fr McGirr, it might equally be that this gift was not the soil per-se but knowledge of the potential of the soil, that is, it was already known by some that the local soil had some sort of healing capabilities (under the right circumstances) but it might take a person with faith in these spiritual practices to enlighten the people as to its true significance.

Some people might also consider that the power of positive belief (or *placebo*) also plays a large role in healing, not just believing that a particular drug will work, but realizing that something is being done to alleviate a particular condition, which is a belief that one might confuse for the true spiritual belief (Schienle et al., 2021).

Of course the choice of the word ‘blessed’ to describe the healing soil also has several connotations including holiness which is thought to derive from the old English, “holy”, meaning, bringing health, healthy or whole. Indeed many spiritual healing practices are concerned with curing the person as a whole rather than one particular part of their health. In the Christian tradition, Jesus instructs an invalid who He had just cured to go and sin no more (John 5:1–15). While this is rather baffling in terms of health, some people interpret this as a call to clean up all parts of his life.

The concept of these holistic considerations has been well-recognized in many ethnopharmacological traditions but perhaps is only just taking root in modern medicine. Part of the problem (with modern medicines) has been that testing of pharmaceutical compounds can become quite complicated if examining more than one compound or one effect. This is what happened in the case of early forms of antibiotics. Researchers would isolate one particular compound which they surmise was the principal active component and use this to treat disease. However, in nature, where many of these pharmaceuticals were originally derived, antibiotics like penicillin or even streptomycin are produced with many other secondary metabolites, some of which are reported to enhance the stability and efficacy of the main pharmacologically active compounds (Terra et al., 2021) such as biosurfactants, reducing agents, pigments, iron chelating compounds and potentiators (of antibiotic activity) (Fukumoto et al., 2008; van Asbeck et al., 1983).

Indeed, the concept of this whole or holistic action and the associated molecular ecology of secondary metabolites has only recently been considered in the search for new types of treatments for drug-resistant bacteria. However, it perhaps illustrates the problems with antimicrobial chemotherapy, where reliance on one component, even if it is a strong component, might not be such a good strategy to avoid resistance. On the other hand a multicomponent approach does not rely on any individual facet but on many factors, so there is less chance of resistance developing (Rabahi et al., 2017).

### Responses to the Discovery of Antibiotic Producing Organisms in the Blessed Clay

Although many people were willing to discuss the issue of the Blessed Clay in a personal capacity, very few were comfortable with being identified. Therefore, in order to respect their anonymity, we have used reports that were printed in other publications. When the research into the Blessed Clay was described to local people in scientific terms by the authors, the explanations were met with a large variation in responses ranging from favorable acceptance to complete rejection (Lidz, 2020). In one instance after a journalist explained the scientific nature of the discovery to one particular parishioner, they replied that “The blessed clay has nothing to do with science. It has to do with faith,” (Baraniuk, 2019). Other parishioners such as the owner of the local public house stated that “the legend of the magical soil is something that’s resonates down through the ages, every town and village in Ireland seems to have a cure that involves taking something from someone and giving it back. Ours mirrors the mysterious side of Irish mythology, all these hidden secrets” (Lidz, 2020).

Although parishioners from the local church where the grave is located were “quietly proud that their little part of the country has received some attention” (Lidz, 2020), there were also negative reactions. Some people saw the discovery of antibiotic producing *Streptomyces* as a curious distraction from religion, even going so far as to describe the healing traditions as promoting paganism (Lidz, 2020). However this can be quite a routine accusation of older healing traditions throughout Ireland. Other parishioners claimed that the visitors to the small countryside church were confusing two stories that were not related. On one hand, there was the story of the antibiotic producing bacteria discovered in the soil from the grave of Fr James McGirr and on the other hand there was the story of the tradition of the healing clay from the grave of Fr James McGirr. They further stated that “the two stories shouldn’t really be linked at all” (Flaherty, 2019). Of course, this is an opinion which is contrary to the main hypothesis of this manuscript.

## Limitations

Discussions of the Blessed Clay of Fr McGirr, which is derived from an oral tradition, are limited by the scarcity of documented material on this subject. Therefore our views represent only a limited insight into these spiritual practices. We also acknowledge that the description of these cures by local people (published in other media) only represent a small demographic of the local population which may or may not be representative of the area as a whole. We are aware that the oral tradition, especially of these spiritual practices is disappearing fast as the population ages. Therefore we recommend that attempts are made to document these practices more thoroughly.

Most importantly, although our discussions reflect on some interesting elements of traditional healing practices associated with soil and spiritual sites, we appreciate that any useful insights or discoveries arising from their analysis should be subject to rigorous scientific tests by the appropriately authorized bodies. Further, our philosophical exploration of the nature of spiritual healing sites is not an endorsement of any particular group or traditional practice, but rather to encourage further scientific research to explore elements that might be useful to modern medicine.

## Conclusions

It is quite clear that the nature of the Blessed Clay, its geography and the surrounding spiritual traditions point to the potential for discoveries in medical science. We therefore think it is imperative that more research is carried out on other sites with similar healing traditions with a view to potentially isolating and identifying microorganisms that produce pharmaceutical compounds. In this way spiritual healing practices might guide new insights in drug discovery.
